# Mental health impact of COVID-19 on healthcare workers versus adults in Africa

**DOI:** 10.4102/phcfm.v16i1.4175

**Published:** 2024-02-29

**Authors:** Mutshidzi Mulondo, Sithembiso Ndlovu, James Ndirangu, Joyce Tsoka-Gwegweni

**Affiliations:** 1 Department of Public Health, Faculty of Health Sciences, University of the Free State, Bloemfontein, South Africa

**Keywords:** COVID-19, mental health, healthcare workers, pandemic, adult general population, Africa

## Abstract

**Background:**

This review mapped the impact of the coronavirus disease 2019 (COVID-19) pandemic on the mental health of healthcare workers (HCWs) and the adult general population in Africa.

**Aim:**

The study focussed on anxiety, depression, post-traumatic stress disorder and suicide cases to determine the impact of COVID-19 on the mental health of the selected population.

**Method:**

A scoping review was conducted on relevant database and search engines. The search resulted in 143 studies. Five studies met the inclusion criteria for synthesis.

**Results:**

Results indicated anxiety was more prevalent among HCWs as opposed to the adult general population, which was in the rise of suicide cases. Among HCWs, mental health was negatively impacted by the loss of their infected patients and concerns over infecting family members. The adult general population was impacted because of isolation and their fear of contracting the virus.

**Conclusion:**

The COVID-19 pandemic led to the increase of mental health issues among HCWs as evidenced by a high prevalence of anxiety compared to that of the adult general population. There was, however, a rise in depression and suicide cases among the adult general population.

**Contribution:**

This study will assist in adding more knowledge to build a robust and responsive strategy to mental health problems during and post-pandemics like COVID-19. Strategies that have appeared effective in combatting the impact of COVID-19 on mental health include support packages established for frontline HCWs such as social media online chat groups.

## Introduction

The coronavirus disease 2019 (COVID-19) was first identified in Wuhan China in 2019 and spread to most countries globally.^1^ The disease was not just seen as an outbreak but was soon declared a global pandemic by the World Health Organization (WHO).^2,3,4^ The pandemic affected both healthcare workers (HCWs) and the adult general population physically, behaviourally, socially and mentally. Furthermore, it brought about economic and medical challenges.^5^ Countries such as China and Italy recorded high cases of infection and deaths among HCWs and regarded them as a high-risk group.^1^

These catastrophic numbers led to the WHO advising countries to follow Non-Pharmaceutical Interventions (NPI) in the absence of a vaccine, which included changes socially, behaviourally and environmentally. These comprised practical changes such as social distancing, wearing of face masks and sanitising.^6^ While the public practised these changes, HCWs continued to provide healthcare services to reduce morbidity and mortality related to COVID-19 infections. Global evidence reveals that HCWs worked under stressful conditions during the COVID-19 pandemic, which affected their mental health status.^7^ The WHO defines mental health as:

[*A*] state of well-being in which the individual realizes his or her own abilities, can cope with the normal stresses of life, can work productively and fruitfully, and is able to make a contribution to his or her community.

A similar definition by Galderisis et al.^8^ defines mental health as:

[*A*] dynamic state of internal equilibrium which enables individuals to use their abilities in harmony with universal values of society; basic cognitive and social skills; ability to recognize, express and modulate one’s own emotions, as well as empathize with others; flexibility and ability to cope with adverse life events and function in social roles; and harmonious relationship between body and mind represent important components of mental health which contribute, to varying degrees, to the state of internal equilibrium. (p. 231)

Research shows that the COVID-19 pandemic was associated with high levels of mental health conditions noted mainly in high-income countries (HICs).^7,9^ There was a notable increase in the risk for depression, anxiety, post-traumatic stress disorder (PTSD) and suicide.^10^ Low- and middle-income countries (LMICs) and resource-constrained contexts were also expected to face the greatest mental health burden associated with the COVID-19 pandemic. This is because research shows that in South Africa, one third of individuals develop a psychiatric disorder during their lifetime.^10^ This is further supported by experts who warned that there could be high levels of depression, suicide and substance use disorders in the world as a result of the COVID-19 pandemic.^11,12^ Among 10 global health issues to track in 2021, WHO included, ‘prevent and treat noncommunicable diseases (NCDs) and mental health conditions’, in which, among other things, they aimed to increase community-based awareness and mental health care.^13^

The bulk of research on the impact of COVID-19 on mental health focussed on the adult general population.^9,12^ Very little is known about the impact of COVID-19 on the Mental health status of HCWs, especially in Africa. Research also shows that in HICs, the COVID-19 pandemic impacted the mental health status of HCWs and adult general population differently.^9^ Evidence reveals that the impact of COVID-19 on HCWs was mainly anxiety, while the adult general population was mainly affected by depression and suicide.^14^ Reasons for the high levels of mental distress among HCWs include increased work demands, unconducive working environment and fatigue.^9^ High-income countries were found to have better developed strategies and resources to address mental health problems among HCWs. In LMICs, strategies to reduce the impact of COVID-19 on the mental health of HCWs are not known.

## Aim

The study focussed on anxiety, depression, PTSD and suicide cases to determine the impact of COVID-19 on the mental health of HCWs and adult general population.

## Objectives

In order to achieve the aim of this study, the following objectives were addressed:

- Identifying the impact of COVID-19 on the mental health of HCWs.
- Identifying the impact of COVID-19 on the mental health of adult general population.
- Comparing the mental health impact of COVID-19 on HCWs versus the adult general population.

## Methods

### Study design

This study employed the methodological framework by Arksey and O’Malley to conduct the scoping review.^11^ The steps followed were: identifying a clear objective(s) to be addressed in this review; identifying relevant literature by conducting a literature search on EBSCOhost and Google Scholar electronic databases; screening of literature to be used in the synthesis and data extraction and recording and reporting the findings of the synthesis and review.

### Literature search strategies

Relevant articles and literature were searched from EBSCOhost and Google Scholar electronic databases; the search engine was set during the early stages of the COVID-19 pandemic from November 2019 until May 2021. An academic search of keywords was conducted using Boolean operators ‘*OR, AND*’ and search strategy, title (TI) (COVID-19 *OR* coronavirus OR 2019-nCoV OR nCoV OR ‘SARS-Cov2’) AND (‘healthcare workers’ OR ‘health care assistan*’ OR ‘general public’ OR ‘adult general population’ OR ‘general populace’ OR ‘Ordinary people’) AND TI (‘mental health’ OR ‘mental stab*’ OR ‘mental-hygiene’ OR ‘mental disab*’). One researcher conducted the electronic search and a librarian assisted in finalising the key term search strategy and obtaining relevant study documents, which were not easily accessible to the researcher.

### Eligibility criteria

The inclusion criteria included all primary research literature published on the mental health impact of COVID-19 on HCWs and the adult general population. Only studies that focussed on the African population and published during November 2019 and May 2021 were included in this review. All study designs with only articles written in the English language were included. The literature that did not meet the above criteria and commentaries and editorials were excluded.

### Identification and selection of studies

**FIGURE 1:**
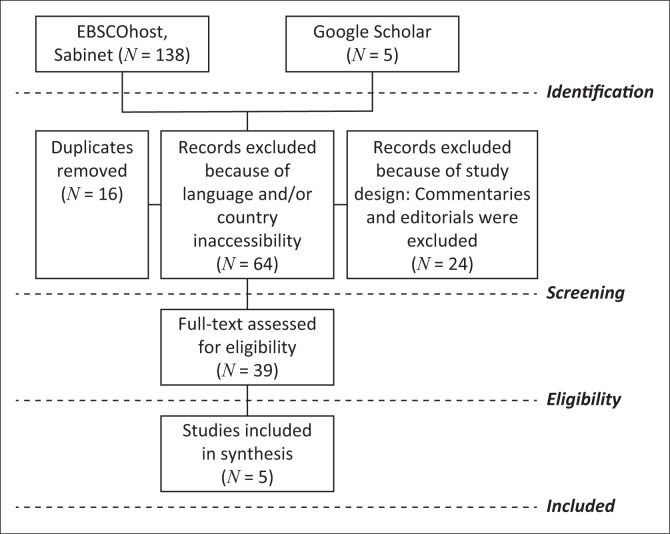
Preferred Reporting Items for Systematic Reviews and Meta-Analyses flow diagram.

Once the librarian had completed the comprehensive literature search, the researchers went through the literature to identify eligible studies. Duplicate studies were excluded from the selected studies. A total of 143 publications were retrieved from EBSCOhost (138) and Google Scholar (5) database platforms. Sixteen (16) duplicate publications were excluded from the review. Of the 127 remaining publications, 88 studies were excluded as they did not meet the inclusion criteria as set out in the study’s eligibility criteria. After reading full texts of the eligible 39studies, 34 more were excluded as they did not meet the methodological or geographical inclusion criteria. Therefore, five publications were included in this review study. This screening process has been indicated in a Preferred Reporting Items for Systematic Reviews and Meta-Analyses (PRISMA) flow diagram as shown in Figure 1.

### Data extraction from selected studies

Once the screening process was completed, the relevant publications were extracted and populated in a data extraction sheet under the domains: author, study design, time period of study, focus, symptoms or signs, risk factor, assessment tool and country (Table 1).

**TABLE 1:** Data extraction sheet.

Author	Study design	Time period of study	Focus (HCW/Public)	Symptoms or signs	Risk Factor	Assessment tool	Country
Kim^8^	Mixed-method	March 2020–May 2020	General adult population	Depressive symptoms; anxiety, financial insecurity, fear of infection and rumination	Childhood trauma	General Health Questionnaire (GHQ-28)	Soweto, South Africa
Htay^15^	Quantitative	April 2020–May 2020	HCWs	60% anxiety, 53% depression, Physical exhaustion; loss of infected patients; personal safety and	Those who stay alone or are single, work in ICU and young professionals	General Anxiety Disorder (GAD-7) and Patient Health Questionnaire (PHQ-9)	Global: 31 LMICs
Ammar et.al.^16^	Quantitative	06 April 2020–11 April 2020	Adult general population: (18 years +)	The COVID-19 home confinement evoked a negative effect on mental wellbeing and emotional status	Those with unhealthy lifestyle behaviours for example poor sleep, unhealthy diet	Mental well-being (Short Warwick-Edinburgh Mental Well-being Scale).	Europe, North Africa, Western Asia and the Americas
El Hayek^17^	Qualitative	November 2019–05 April 2020	Adult general population: geriatrics	High prevalence of depression in those with Parkinson’s disease, dementia of Alzheimer’s type and vascular dementia.	Living alone, living in a nursing home, illiteracy and having chronic diseases are associated with geriatric depression.	-	Arab countries in the Middle East and North Africa
Gloster^18^	Quantitative	07 April 2020 and 07 June 2020	Adult general population	Low levels of mental health to moderate mental health	Social support, education level and psychologically flexible (versus rigid) responding.	Stress = Perceived Stress Scale; Depressive symptomatology = disengagement subscale of the Multidimensional State Boredom Scale.	An international study. 78 countries.

ICU, intensive care units; HCW, healthcare workers; LMIC, low- and middle-income countries; COVID-19, coronavirus disease 2019.

### Data analysis

The data were analysed using descriptive analysis for the five studies that were included in the review. In the first analysis, the studies were described by study population, region or country and study design. A second analysis described the impact of COVID-19 on each study population. This was done by identifying the mental health conditions that affected each of the HCWs and adult general population as well as the associated risk factors. Where data was available, the prevalence for each condition for each study population was described. The final analysis compared the impact of COVID-19 on mental health between HWCs and the general population by conditions and their risk factors.

### Ethical considerations

Ethics waiver was obtained from the Health Sciences Research Ethics Committee, University of the Free State (reference number UFS-HSD2021/0501/2505). There were no human participants, and therefore no consent was violated.

## Review findings

### Description of the reviewed studies

The five studies that were included in the review were conducted in South Africa; 31 global countries including 10 sub-Saharan African countries (Ethiopia, Kenya, Lesotho, Mozambique, Nigeria, Rwanda, South Africa, Tanzania, Uganda, Zimbabwe), globally including North Africa and globally including four Southern African countries (Angola, South Africa, Namibia and Uganda). One study used mixed methods (study 1), while the others used either quantitative (studies 2, 3 and 5) or qualitative study designs (study 4). In terms of the study population, four studies focussed on the adult general population and one studied on HCWs (Table 2).

**TABLE 2:** Description of the reviewed studies.

Study	Study population	Region (Country)	Study design
1	General adult population	Southern Africa (South Africa)	Mixed methods
2	HCWs	31 global countries (including 10 African countries: Ethiopia, Kenya, Lesotho, Mozambique, Nigeria, Rwanda, South Africa, Tanzania, Uganda, Zimbabwe	Quantitative
3	General adult population	North Africa Western Asia	Quantitative
4	General adult population	North Africa Middle East	Qualitative
5	General adult population	78 global countries (including Southern Africa: Angola, South Africa, Namibia, Uganda)	Quantitative

HCW, healthcare workers.

### Summarising and comparing the findings on mental health impact of coronavirus disease 2019

The conclusions from the data extraction sheet were summarised and compared under the domains: HCW mental health impact of COVID-19, adult general population mental health impact of COVID-19 and HCW mental health impact versus adult general population mental health impact of COVID-19.

From the five studies included in this review, one study looked at HCWs’ mental health impact of COVID-19, four looked at the adult general population mental health impact of COVID-19. It is reported that in the 2097 HCWs recruited from 31 LMICs, the prevalence of anxiety was 60% and depression was 53%, which were mainly in mild and moderate levels. These conditions are reported to have emerged as a result of physical exhaustion, loss of infected patients, concern for personal safety and passing of the COVID-19 infection to their loved ones (Table 3).

**TABLE 3:** Impact of coronavirus disease 2019 on mental health.

Group	Mental health condition	Risk factors
HCWs	60% anxiety 53% depression Physical exhaustion	Loss of COVID-19-infected patients Concern for personal safety and passing of the COVID-19 infection to their loved ones Staying alone Single, Work in ICU, Young professionals
Adult general population	Depression, anxiety, fear of infection, rumination	Childhood trauma
Adult general population	-	Home confinement during lockdown, unhealthy lifestyle behaviours (poor sleep and unhealthy diet)
Adult general population	High prevalence of depression	Dementia, Parkinson’s disease, living alone, living in a nursing home, chronic diseases
Adult general population	Low-moderate levels of mental health	Social support, education level, psychologically flexible,

HCW, healthcare workers; COVID-19, coronavirus disease 2019; ICU, intensive care unit.

Among the adult general population, a higher perceived risk of COVID-19 infection was associated with greater depressive and anxiety symptoms in individuals. It is reported that these mental health conditions were accompanied by a five-fold increase in the number of suicide cases from 418 before the pandemic to 2 114 during the pandemic because of joblessness. In South Africa, it is indicated that suicide cases were further exacerbated by the low numbers of mental healthcare service usage and limited access. It is reported that only 27% of patients with severe mental illness received treatment and there were only 0.31 psychiatrist per 10 000 uninsured population (Table 3). The mental health distress among the adult general populations is said to be related to financial insecurity, fear of contracting infection, rumination and isolation. The risk factors identified were childhood trauma, poor diet and sleep, lack of social support, low levels of education and psychological inflexibility, living alone or in a nursing home or existence of chronic diseases (Table 3).

## Discussion

### Impact of coronavirus disease 2019 pandemic on mental health of healthcare workers

The results of this study indicate a significant impact of COVID-19 on the mental health of HCWs. According to Xiong et al.,^4^ there has been a rise in psychological distress among HCWs. This is also evidenced in Htay et al.^16^ global perspective study, which included 2097 HCWs from 31 LMICs and revealed that HCWs’ mental health was impacted by the COVID-19 pandemic. Another study agreed with Htay et al.^16^ that HWCs were mainly impacted by the COVID-19 pandemic because of loss of their infected patients, concern over their own personal safety and fear of passing infections to family members.^3^ A different study showed that battling COVID-19 in the frontlines made HCWs vulnerable to psychological distress with them exhibiting high levels of depression, stress, anxiety, distress, anger, fear, insomnia and PTSD.^1^ These findings support those from previous outbreaks such as the Middle East Respiratory Syndrome (MERS) in 2006 and the Severe Acute Respiratory Syndrome (SARS) in 2011, which showed that frontline HCWs were afraid of stigmatisation in addition to the fear of infecting family members.^1^ This further contributed to their psychological distress.

### Impact of coronavirus disease 2019 pandemic on mental health of the adult general population

According to Kim et al.,^10^ higher perceived risk of COVID-19 infection was associated with greater depressive symptoms in individuals. There was markedly an increase in the number of suicide cases as shown in a Canadian study with a five-fold increase of suicide cases during the pandemic as a result of joblessness.^4^ In South Africa, suicide cases were further exacerbated by the low numbers of usage and limited access to public mental healthcare.^10^

### Healthcare workers mental health impact versus adult general population mental health impact

The findings of this study indicate that the impact on HCWs’ mental health has relatively been similar to the impact on the adult general population.^3^ Furthermore, this impact has been traced back to previous epidemics such as SARS in which both HCWs and the adult general population displayed signs of anxiety. Both HCWs and adult general population were plagued by concerns and fears of infecting their family members and loved ones. In addition to loved ones, HCWs had the added weight of being concerned about their patients, long working hours as well as media scrutiny.^3^

Among the adult general population, adults such as parents have experienced the pandemic differently, for example, they have had to endure role confusion as they had to double as both care providers and home-school teachers.^5^ This has led to higher parenting dissatisfaction and lower distress tolerance.

Although suicide cases appear to have been neglected before COVID-19 as they were not necessarily in the spotlight, these cases appear to have remained equal and neutral during the pandemic because of the social support received by individuals speedily.^5^ However, that may be because of the scarce data from previous pandemics. Nonetheless, depression that was noted as a response to loss was precipitated during COVID-19 primarily because of the loss of loved ones.^5^

## Recommendations

The findings of this study reveal that research on mental health has mostly focussed on the adult general population. This further highlights the need for more research to be conducted on the HCWs to understand the mental health impact of the COVID-19 pandemic on them. More studies should be conducted to determine the impact of COVID-19 in the latter part of pandemic as some people may still be experiencing the impact post-pandemic.

Increased psychological difficulties during the COVID-19 pandemic were from stress, which is when situational demands outweigh one’s real or perceived resources to address them.^5^ World Health Organization has emphasised, during the COVID-19 pandemic, the importance of mental health support for HCWs. Mental health support for HCWs should be made available particularly for those who stay alone or are not in intimate relationships and are young professionals in their field.^16^

Workplace tele-counselling or hotline mental health support from external organisations should be implemented among HCWs as it has proven a success in other countries. This includes digital mental health support packages established for frontline HCWs and peer support project led by the mental health professionals, which uses social media online chat groups in the United Kingdom. Peer support projects can also be achieved through the increase of community-based awareness and advocacy for mental health.^8,16^ The world of Fourth Industrial Revolution (4IR) should be embraced in dealing with mental health issues, particularly during crises that necessitate isolation.

Future research should be conducted to explore the role of religion in coping with the psychological burden during the pandemic.^16^ Spirituality may be beneficial in combating psychological distress and its benefit should therefore be explored further.

## Conclusion

Both HCWs and the adult general population had their mental health impacted by pandemic owing to different situational reasons. The fear of infecting family members and loved ones appeared to be a common thread for both HCWs and the adult general population, which induced other mental health issues. For HCWs, mental health issues such as anxiety were mostly because of the high loss of patients. For the adult general population, increased cases of anxiety were mainly because of the fear of the unknown and increased domestic responsibilities. The isolation from significant others for both HCWs and the adult general population necessitates digital interventions to combat the mental health challenges in future devastations. This can be in the form of tele-counselling as digital mental health support packages and peer support projects through social media platforms.
